# Construction of a Nutrition Management Guidance Flowchart for Patients With Chronic Wounds Based on Best Evidence

**DOI:** 10.1111/iwj.70172

**Published:** 2025-03-25

**Authors:** Huan Liu, Chunxiao Liu, Donghua Ma, Xuefen Shen, Yanmin Lu, Chenxi Pu, Jiamin Meng, Ming Cheng

**Affiliations:** ^1^ Department of Nursing, College of Medicine, The First Affiliated Hospital Zhejiang University Hangzhou China; ^2^ Department of Nutrition, College of Medicine, The First Affiliated Hospital Zhejiang University Hangzhou China; ^3^ Department of Orthopedic Surgery, College of Medicine, The First Affiliated Hospital Zhejiang University Hangzhou China

**Keywords:** best evidence practice, chronic wounds, clinical practice, evidence based, nutrition management

## Abstract

The lack of systematic and comprehensive clinical nutrition management practices for patients with chronic wounds necessitates the construction of a scientifically‐based, standardised, normalised nutrition management guidance procedure for these patients in clinical settings. The purpose of this study was to: (i) summarise the best evidence for nutrition management in patients with chronic wounds by performing a systematic literature search and rigorous evaluation, and (ii) construct a nutrition management guidance flowchart for these patients based on best evidence. We reported the best evidence summary for nutrition management in patients with chronic wounds by following the Evidence Summary Reporting Standard of Fudan University Evidence‐Based Care Center. An expert panel was established to construct a flowchart of nutrition management guidance for patients with chronic wounds by discussing existing evidence entries. After the quality evaluation, 17 studies (5 guidelines and 12 systematic reviews) were included, which provided extractable data for this summary of evidence. The best evidence of nutrition management in patients with chronic wounds was summarised, and a nutrition management guidance flowchart based on this was constructed, which can provide references for medical staff to guide nutrition management for patients with chronic wounds. Looking ahead, more high‐quality research is needed to focus on specific personalised nutrition management programmes for patients with chronic wounds.

**Summary**

- The guidance flowchart is clear, easily embraced by medical personnel and patients and promotes scientific, standardised and normalised nutrition management among patients with chronic wounds in clinical settings.
- The best evidence of nutrition management includes four aspects: nutrition assessment and diagnosis, nutrition care plan, nutrition education and counselling and multidisciplinary teamwork.
- Healthcare workers should complete nutrition screening, primary assessments and comprehensive nutrition assessment for patients with chronic wounds sequentially, identify different levels of nutrition diagnosis and adjust nutrition management strategies in a timely manner.
- Nutrition education is advised for all patients with chronic wounds and their caregivers.

## 1 Introduction

Chronic wounds are those that fail to heal in a timely and orderly manner through ‘standard treatment’ and are caused by a variety of pathological conditions, such as diabetes, vascular dysfunction, skin pressure, or infections [1]. There is a high incidence of chronic wounds among people around the world, causing a significant economic burden [2]. A survey in China showed that individuals with chronic wounds faced an average healthcare cost of 55 270 RMB and were hospitalised for at least 21 days [3].

Nutrition plays a vital role in the healing of chronic wounds. Adequate nutrition is essential for tissue repair and wound healing [4]. Malnutrition can not only delay the healing process and increase the risk of the development of a non‐healing wound but also may result in unfavourable clinical outcomes for patients with chronic wounds [5, 6, 7]. Earlier research has shown that malnutrition can increase the risk of infection [7], hinder the body's functional recovery, worsen the quality of life, prolonged hospital stays and even increase the risk of death [8]. In fact, patients with chronic wounds may be at a higher risk of malnutrition than other populations due to various factors (e.g., wound inflammation and infections and increased demand for nutrition) [9]. Studies have reported that the prevalence of malnutrition in patients with chronic pressure ulcers and diabetic foot ulcers is as high as 72.3% and 75% when considering multiple comorbidities and medications, respectively, and only 4.5% of patients with chronic wounds maintain standard levels of nutrition [10]. Consequently, to maintain a proper nutritional status in patients with chronic wounds, it is important to develop scientifically based and effective nutrition management strategies.

Despite the multitude of pathogenic factors of chronic wounds, different types of chronic wounds follow similar healing mechanisms and processes [11], suggesting that patients with chronic wounds might have similar nutrient requirements. A growing body of evidence shows that the effectiveness of wound healing depends on nutrition interventions [4, 12]. Some studies have highlighted that Vitamins D and C could promote the growth of epithelial cells, fibroblasts and granulation tissues, contributing to wound healing [13, 14]. In addition, a deficiency of amino acids and minerals, such as copper and zinc in wound patients, may hinder tissue cell regeneration [15]. Nonetheless, the nutrition interventions provided in these articles were not comprehensive, and the quality of these studies is inconsistent. Several expert consensus and systematic reviews exist relating to the nutrition management of patients with chronic wounds [16, 17, 18, 19], but these are lengthy and difficult to understand, making it challenging for healthcare professionals to access and implement useful information in their clinical work. For example, Apergi, Dimosthenopoulos, and Papanas [19] reviewed the frequent dietary assessments and nutrition education of patients with diabetic foot ulcers and found a lack of specific methods for evaluating patient nutrition. Furthermore, most nutrition management studies did not summarise the level of evidence and the intensity of the recommendations, which was not conducive to further clinical transformation and the scientific application of evidence. The summary of best evidence is intended to provide a comprehensive overview of available data on a specific research query or to collect research questions related to a singular subject [20]. It is recommended that priority is given to searching for and including evidence that has been rigorously evaluated for quality, such as guidelines, systematic reviews and expert consensus to guide clinical practice [20]. Finally, the establishment of a transparent nutrition management process is necessary to promote scientific and standardised nutrition management for patients with chronic wounds.

Taken together, effective nutrition management is crucial in promoting wound healing and improving the clinical outcomes of patients with chronic wounds. However, an evidence‐based nutrition management process has not been developed for such patients. In line with this demand, the purpose of this study was to: (i) summarise the best evidence for nutrition management in patients with chronic wounds through a systematic literature search and rigorous evaluation, and (ii) construct a nutrition management guidance flowchart for these patients based on best evidence.

## 2 Materials and Methods

Based on the evidence aggregation generation methodology, Fudan University Evidence‐Based Nursing Center has developed a reporting standard [20], with the following reporting criteria: (i) establishment of the problem; (ii) literature screening and evaluation; (iii) summary and grading of evidence; and (iv) development of practical recommendations. In line with the Evidence Summary Reporting Standard of Fudan University Evidence‐Based Care Center [20], this study reports the best evidence summary for nutrition management in patients with chronic wounds. An expert panel was established to construct a flowchart of nutrition management guidance for patients with chronic wounds by discussing existing evidence entries [21].

### 2.1 Establishment of the Problem

The PIPOST model was proposed by Fudan University Center for Evidence‐Based Nursing to report the establishment of the problem (see Table 1).

**TABLE 1:** PIPOST model to report the establishment of the problem.

PIPOST items	Content
First P (population)	Refers to the target population for evidence application, which includes patients with chronic wounds who are over 18 years of age
I (intervention)	Focuses on content related to nutrition management, such as nutrition risk screening, assessment, diagnosis, intervention, monitoring, etc.
Second P (professional)	Refers to the medical staff in the hospital, who are professionals in applying evidence‐based practices
O (outcome)	Pertains to health‐related outcomes, such as wound healing, quality of life, mortality, readmission rates and economic impacts
S (setting)	Denotes the location where evidence‐based practices are applied, which can be either in a hospital setting or within the community
T (type of evidence)	Clinical guidelines, best decision‐making, expert consensus, systematic review and evidence summary

### 2.2 Literature Screening and Evaluation

#### 2.2.1 Evidence Sources and Retrieval Strategy

Based on the ‘6S’ evidence resource pyramid model, the search began at the top of the pyramid through a computerised decision support system and gradually descended towards the pyramid [20]. After consulting with an expert on library electronic databases, a researcher developed a search strategy, which was then carried out by two other reviewers who had undergone evidence‐based training. The following databases were screened for published eligible studies (from inception until 19 November 2023): UpToDate, BMJ Best Practice, the Cochrane Library, Australian Joanna Briggs Institute (JBI) Evidence‐Based Medicine Center Healthcare Database, National Institute for Health and Clinical Excellence (NICE), Guidelines International Network (GIN), National Guideline Clearinghouse (NGC), Scottish Intercollegiate Guidelines Network (SIGN), Embase, PubMed, CINAHL, SinoMed, China National Knowledge Infrastructure (CNKI), Wanfang Data Knowledge Service Platform and VIP Database.

The keywords used were ‘chronic wounds’ (chronic refractory wounds, refractory wounds and skin ulcers) and ‘nutrition’ (nutrition intervention, nutrition screening, nutrition diagnosis, diet, dietary, oral and food). No language restrictions were applied in the literature search. The reference lists of included articles and grey literature sources were also reviewed to identify potentially eligible studies. Retrieved articles were checked and managed with the help of EndNote software (Version X9; Clarivate Analytics, Philadelphia, PA).

#### 2.2.2 Inclusion and Exclusion Criteria

The inclusion criteria were as follows: (i) The study population included adult patients with chronic wounds. Chronic wounds were defined as those that, despite a month of ‘standard treatment’, showed no notable enhancement in healing (Xiaobing Fu et al. ‘standard diagnosis of chronic wounds’) [22]; (ii) literature focusing on nutrition management strategies (including nutritional risk screening, assessment, diagnosis, intervention and monitoring); (iii) literature types included clinical guidelines, best decision‐making, expert consensus and systematic review; (iv) the latest versions of updated or revised clinical guidelines; and (v) English and Chinese literature only.

The exclusion criteria were as follows: (i) duplicate publications, translated literature or literature that consists a plan, draft or only an abstract; (ii) guideline interpretations, conference abstracts or literature with incomplete information that could not be accessed in full; (iii) non‐English or non‐Chinese literature; and (iv) literature of low quality.

#### 2.2.3 Selection Process

First, two researchers independently conducted an initial screening of the articles by reading the titles and abstracts to identify eligible studies according to pre‐established inclusion and exclusion criteria. Subsequently, another two researchers independently read the entire text to identify the articles to be included. In case of any discrepancies, they consulted with a third researcher to reach a consensus. The reasons for exclusions during the comprehensive text screening process were documented. Finally, after a thorough review of the entire literature, the selection of articles was completed.

#### 2.2.4 Literature Quality Appraisal

The appropriate assessment tools were selected based on the type of literature. For best practices and evidence summaries, the original literature was consulted that presented the evidence.

The methodological quality of guidelines was assessed using the Appraisal of Guidelines for Research & Evaluation Instrument II (AGREE II), which includes six domains: scope and purpose, stakeholder involvement, rigour of development, clarity of presentation, applicability and editorial independence, comprising a total of 23 items [23]. Each item was scored on a 7‐point Likert scale from 1 (strongly disagree) to 7 (strongly agree). The final score for each domain was calculated using a standardised percentage. The calculation is as follows: (obtained score − least possible score)/(maximum possible score − least possible score) × 100%. The results of the guideline evaluation are divided into three recommended levels: A level (recommended) with scores of ≥ 60% in all six domains; B level (recommended, but needs modification) with scores ≥ 30% in more than three domains, but some domains are < 60%; and C level (not recommended) if there are < 30% in three or more domains. According to the manual requirements, two reviewers independently rate each item in AGREE II, to ensure consistency in understanding the evaluation items and guidelines [23]. Before starting the evaluation, one guideline is randomly selected for pre‐evaluation, and the evaluation results are thoroughly discussed.

The methodological quality of systematic reviews was assessed using the JBI Critical Appraisal Checklist for Systematic Reviews and Research Syntheses [24]. The tool consists of 11 items from the perspectives of evidence‐based questions, search strategy, quality assessment of the literature, extraction and synthesis of information, combining research methods and publication bias. Every item is categorised as ‘yes’, ‘no’, ‘unclear’ or ‘not applicable’. Following this system, a total score was calculated for each systematic review [24]. Reviews excluded literature if they did not achieve a score of at least 50% on critical appraisal, the pre‐established cut‐off score agreed upon by the researchers.

### 2.3 Summary and Grading of Evidence

Two researchers with training in evidence‐based medicine, along with specialised nurses in the wound care field, extracted and summarised the evidence based on the following principles: [20] (i) when the content was consistent, the evidence supporting the condensation of specialty was screened; (ii) when contents from multiple sources were similar or complementary, they were merged into one piece of evidence; and (iii) when the contents from multiple sources conflicted, the evidence with the highest quality or most recently published evidence in an authoritative evidence‐based journal was included. The extracted contents included basic information from the literature, such as the author, the literature's subject, publication date, information source and literature category. The JBI Evidence‐Based Healthcare Centre's 2014 version was utilised to categorise the evidence classified into Levels 1–5, where Level 1 indicates the highest grade and Level 5 denotes the lowest grade [24].

### 2.4 Development of Practical Recommendations

An expert group was established, consisting of seven experts with ≥ 5 years of experience in the field of chronic wounds and two experts in the field of nutrition. An expert meeting was held to validate the strength of the recommended evidence, which was then divided into strong (A) and weak (B) recommendations. If experts disagreed on an evidence item, consensus was reached with at least 2/3 of the experts agreeing.

### 2.5 Construction Guidance Flowchart

Taking into account the best evidence items, actual diagnosis and treatment protocols, variations among patients with chronic wounds and the simplicity and practicality of clinical application, the expert panel held three deliberations to ultimately develop a flowchart for the nutrition management of chronic wounds.

## 3 Results

### 3.1 Literature Review and General Information

**FIGURE 1:**
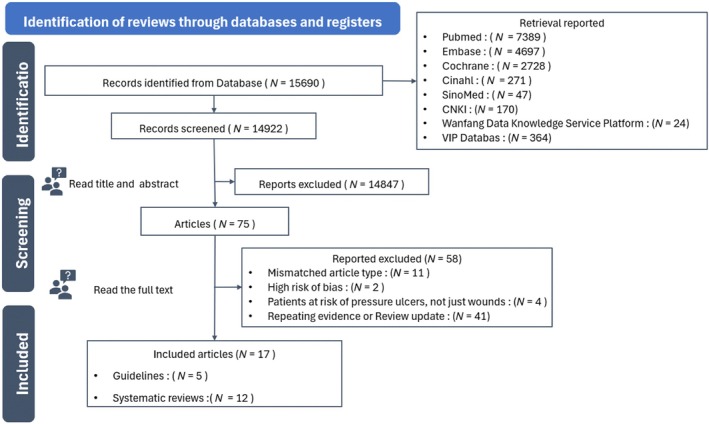
Flowchart search and review selection process.

The PRISMA flowchart of the literature retrieval procedure is shown in Figure 1. The electronic search yielded 15 690 records. After assessing the titles and abstracts, 75 articles failed to meet the inclusion criteria (mismatched article types, *N* = 11; low article quality, *N* = 2; study subjects were healthy people, not just wounds, *N* = 4; repeating evidence or review update, *N* = 41), yielding 17 studies that provided extractable data for this summary of evidence (guidelines, *N* =  5; systematic reviews, *N* = 12) [15, 16, 17, 19, 25, 26, 27, 28, 29, 30, 31, 32, 33, 34, 35, 36, 37].

General information of the included literature is presented in Table 2. Most of the evidence focuses on chronic wounds, mainly diabetic foot ulcers, pressure injuries and vascular wound populations, with less information about trauma and burns leading to chronic wounds. These studies have explored various nutrition management approaches, such as nutrition assessment, nutrients interventions and nutrition health education.

**TABLE 2:** General information of the included literature (*N* = 12).

Author (year)	Study location	Literature source	Type	Content
Chen et al. (2023) [25]	Australia	PubMed	Guideline	Guidelines on interventions to enhance healing of foot ulcers in people with diabetes (IWGDF 2023 update)
Munoz and Posthauer (2022) [26]	Europe	Embase	Guideline	Nutrition strategies for pressure injury management
American Limb Preservation Society (2022) [27]	The USA	SinoMed	Guideline	Nutrition intervention in adults with diabetic foot ulcers
Peng (2019) [16]	China	CNKI	Guideline	Medical nutritional treatment of diabetic foot ulcers
Chinese Medical Association (2021) [17]	China	CNKI	Guideline	Prevention and treatment of type 2 diabetes
Kurian et al. (2023) [28]	India	PubMed	Systematic review	The relationship between micronutrient levels and diabetic foot ulcers
Apergi, Dimosthenopoulos, and Papanas (2023) [19]	Greece	Embase	Systematic review	The effects of dietary nutrition traits on the treatment of diabetic foot ulcers
Daher et al. (2022) [29]	The USA	Embase	Systematic review	Oral nutritional supplements and wound healing
Bechara et al. (2021) [30]	Australia	Embase	Systematic review	The impact of nutrition on diabetic foot ulcers
Saeg et al. (2021) [31]	The USA	Embase	Systematic review	The impact of nutrition on wounds
Moore, Corcoran, and Patton (2020) [32]	Ireland	Embase	Systematic review	Nutritional intervention of diabetic foot ulcers
Zhao et al. (2018) [15]	China	CNKI	Systematic review	The impact of enteral nutrition supplements on the prevention and treatment of pressure ulcers
Liu, Shen, and Chen (2017) [33]	China	Embase	Systematic review	The effects of arginine‐rich enteral nutrition in the treatment of pressure ulcers
Ye and Mani (2016) [34]	China	PubMed	Systematic review	Nutritional supplementation for chronic wounds on the lower limbs
Guo et al. (2016) [35]	China	CNKI	Systematic review	The effect of enteral nutrition on pressure wounds
Blanc et al. (2015) [36]	Brazil	Embase	Systematic review	The effect of enteral nutrition on pressure ulcers
Langer and Fink (2014) [37]	Germany	PubMed	Systematic review	Nutritional intervention for preventing and treating pressure ulcers

### 3.2 Literature Quality Evaluation Results

The methodological quality assessment and the recommendation level of five guidelines are presented in Table 3. Two guidelines received a B‐level recommendation, and three guidelines were labelled as C‐level recommendations due to their lack of rigour in development. Essentially, the articles did not explicitly explain details of the process of retrieving evidence and the criteria for selecting evidence. The methodological quality assessment of 12 systematic reviews is presented in Table 4. Overall, the total scores of the included systematic reviews ranged from 6 to 11 points, indicating high quality.

**TABLE 3:** AGREE II scores of the included guidelines (*N*  = 5).

Study	Domain 1	Domain 2	Domain 3	Domain 4	Domain 5	Domain 6	≥ 60%	≤ 30%	Quality evaluation
	Scope and purpose (%)	Stakeholder involvement (%)	Rigour of development (%)	Clarity of presentation (%)	Applicability (%)	Editorial independence (%)			
Chen et al. (2023) [25]	83.33	91.67	93.75	100.00	54.17	100.00	5	1	B
Munoz and Posthauer (2022) [26]	88.89	75.00	67.71	100.00	33.33	50.00	4	2	B
American Limb Preservation Society (2022) [27]	50.00	50.00	31.25	38.89	37.50	75.00	1	5	C
Chinese Medical Association (2021) [17]	50.00	22.22	12.50	66.67	29.17	50.00	1	5	C
Peng (2019) [16]	50.00	66.67	14.58	33.33	20.83	25.00	1	5	C

**TABLE 4:** Quality evaluation results of systematic review (*n* = 12).

Author (year)	Item 1	Item 2	Item 3	Item 4	Item 5	Item 6	Item 7	Item 8	Item 9	Item 10	Item 11	Total points
Kurian et al. (2023) [28]	Yes	Yes	Yes	Yes	Yes	Yes	Yes	Yes	Yes	Yes	Yes	11
Apergi, Dimosthenopoulos, and Papanas (2023) [19]	Yes	Yes	Yes	Yes	Unclear	Unclear	Unclear	Na	Unclear	Yes	Yes	7
Daher et al. (2022) [29]	Yes	Yes	Yes	Yes	Yes	Yes	Yes	Na	Unclear	Yes	Yes	9
Bechara et al. (2021) [30]	Yes	Yes	Yes	Yes	Unclear	Unclear	Unclear	Na	Unclear	Yes	Yes	6
Saeg et al. (2021) [31]	Yes	Yes	Yes	Yes	Unclear	Yes	Yes	Na	Unclear	Yes	Yes	8
Moore et al. (2020) [32]	Yes	Yes	Yes	Yes	Yes	Yes	Yes	Yes	Yes	Yes	Yes	11
Zhao et al. (2018) [15]	Yes	Yes	Yes	Yes	Yes	Yes	Yes	Yes	Unclear	Yes	Yes	10
Liu, Shen, and Chen (2017) [33]	Yes	Yes	Yes	Yes	Yes	Yes	Yes	Na	Unclear	Yes	Yes	9
Ye and Mani (2016) [34]	Yes	Yes	Yes	Yes	Yes	Yes	Yes	Yes	Unclear	Yes	No	9
Guo et al. (2016) [35]	Yes	Yes	Yes	Yes	Yes	Yes	Yes	Yes	Unclear	Yes	Yes	10
Blanc et al. (2015) [36]	Yes	Yes	Yes	Yes	Yes	Yes	Yes	Na	Unclear	Yes	Yes	9
Langer and Fink (2014) [37]	Yes	Yes	Yes	Yes	Yes	Yes	Yes	Yes	Yes	Yes	Yes	11

*Note:* Item 1: Is the review question clearly and explicitly stated? Item 2: Were the inclusion criteria appropriate for the review question? Item 3: Was the search strategy appropriate? Item 4: Were the sources and resources used to search for studies adequate? Item 5: Were the criteria for appraising studies appropriate? Item 6: Was critical appraisal conducted by two or more reviewers independently? Item 7: Were there methods to minimise errors in data extraction? Item 8: Were the methods used to combine studies appropriate? Item 9: Were the methods used to combine studies appropriate? Item 10: Were recommendations for policy and/or practice supported by the reported data? Item 11: Were the specific directives for new research appropriate?

### 3.3 Guidance Flowchart Based on Evidence

**FIGURE 2:**
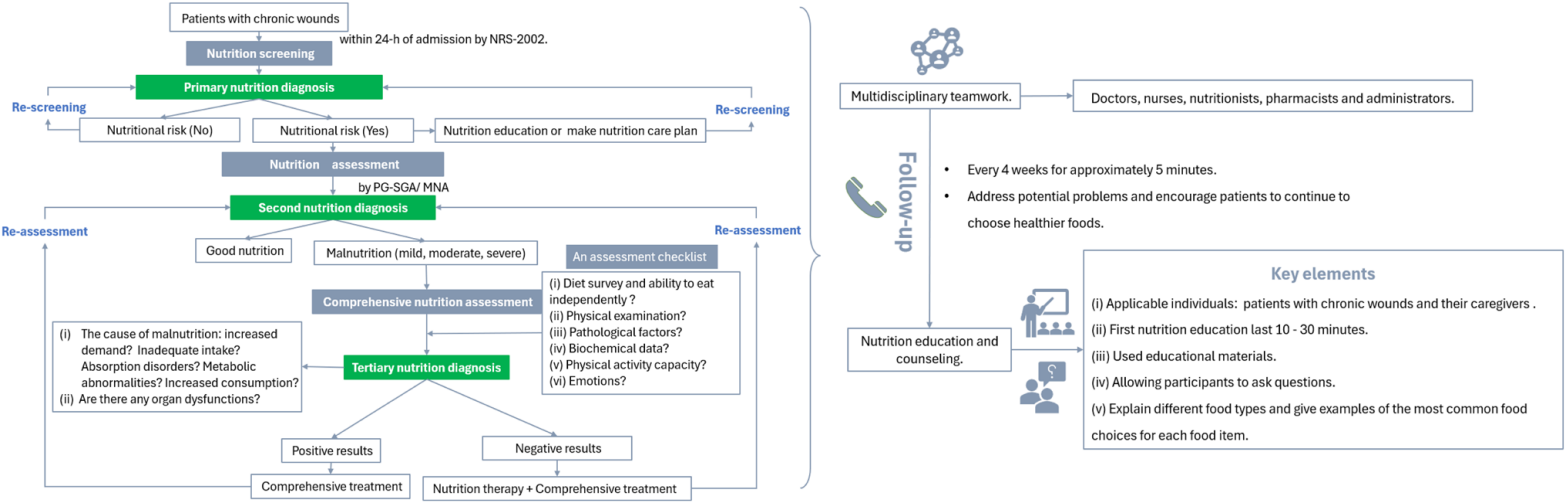
A nutrition management guidance flowchart for patients with chronic wounds based on evidence.

Table 5 shows 26 pieces of evidence on nutrition management strategies in patients with chronic wounds, considering four aspects: nutrition assessment and diagnosis, nutrition care plan, nutrition education and counselling and multidisciplinary teamwork. A nutrition management guidance flowchart for patients with chronic wounds based on evidence is presented in Figure 2.

**TABLE 5:** Summary of evidence for nutrition management in patients with chronic wounds.

Category	Content of evidence	Level	Recommend level
*(i) Nutrition assessment and diagnosis*			
1	All patients with chronic wounds should undergo nutrition screening within 24 h of admission using the Nutritional Risk Screening 2002 (NRS 2002) [17, 38] Primary nutrition diagnosis: Is there nutrition risk? Yes (total scores ≥ 3) □ No (total scores < 3) □	5b	A
2	Patients at nutritional risk should determine the risk of malnutrition and establish a secondary nutrition diagnosis [17, 27] (1) Patient‐Generated Subjective Global Assessment (PG‐SGA) is recommended for general patients, and the Mini Nutritional Assessment (MNA) is recommended for elderly patients with chronic wounds [17] Secondary nutrition diagnosis: Good nutrition □ Malnutrition (mild, moderate and severe) □	5b	A
3	Patients with malnutrition should determine the causes and severity of malnutrition and assess its impact on organs by a comprehensive nutrition assessment [17, 27, 38] (1) Diet survey and ability to eat independently [38] What is the average daily dietary intake of patients? □ Can patients who live alone cook? □ Can patients get enough food? □ Does dietary restriction affect a patient's ability to maintain a balanced diet? □ Do individuals have difficulty swallowing, affecting intake and weight? □ (2) Physical examination [17] Is the patient's weight within the normal range? Has there been an unintentional weight loss of 5% within 30 days or 10% within 6 months? [17] □ Are there any symptoms such as muscle atrophy, skin swelling or organ swelling, hair loss? □ Have medical examinations verified malnutrition? □ (3) Pathological factors Whether any medical interventions, such as chemotherapy, radiation therapy or dysphagia examinations, could affect an individual's nutritional status? □ Did the patient take any medication that could cause weight gain or loss? □ (4) Do biochemical data indicate nutrition concerns, such as low protein content (albumin, prealbumin, etc.), anaemia (haemoglobin, transferrin, etc.), dehydration or uncontrolled diabetes? Are the individual's laboratory values current? [38] □ (5) Physical activity capacity: Mobility and fall frequency? □ (6) Are emotions affecting nutritional status, such as anxiety and depression? [17] □ Tertiary nutrition diagnosis. The cause of malnutrition: Increased demand? Inadequate intake? Absorption disorders? Metabolic abnormalities? Increased consumption? □ Are there any organ dysfunctions? □	5b	A
4	The continuous diagnosis and treatment mode of risk screening, evaluation, diagnosis, intervention and monitoring should be implemented clinically	5b	A
*(ii) Nutrition care plan*			
5	Patients at risk of malnutrition or with malnutrition should intake 30–35 kcal/kg of energy per day [38]	1a	A
6	Patients at risk of malnutrition should consume 1.25–1.5 g/kg of protein per day and adjust their protein supply ratio to focus on the availability of quality protein [17, 25, 38]	1b	A
7	The intake ratio of long‐chain saturated fatty acids, monounsaturated fatty acids and polyunsaturated fatty acids should be maintained at 1:1:1, with specific supplementation of omega‐3 fatty acids in the short term [16]	1b	A
8	Monitor micronutrient levels and appropriately add micronutrients to groups deficient in these nutrients, under the supervision of an expert doctor and nutritionist [28, 29, 30, 31, 32, 37]. (1) Vitamin A supplementation (10 000–25 000 units); (2) Multivitamin B group (B1, B6, B9 and B12); (3) Vitamin C supplementation of 500 mg/day for patients with simple wounds and 2 g/day for patients with complex wounds; (4) supplements of zinc, copper, selenium, calcium and magnesium may facilitate the healing of wounds (including diabetic foot ulcer, pressure injury, burn and traumatic wounds); (5) personalised oral and/or intravenous administration in combination with patient blood routine and iron metabolism test results	1b	A
9	Most patients with chronic wounds are recommended to have a minimum liquid consumption of 1 mL/kcal/day [17]	5b	B
10	Patients with chronic wounds suffering from malnutrition and various comorbidities, or those who are older, should receive oral nutritional supplements rich in calories and proteins, with an energy density ranging from 1.5 to 2.4 kcal/mL, between meals for a minimum of 4 weeks [15, 29, 37]	1b	A
11	Oral nutritional supplements, which contain arginine, glutamine, B‐hydroxy‐B‐methylbutyrate, essential minerals like zinc, magnesium, Vitamin A, Vitamin B9, Vitamin D, Vitamin E, antioxidants and other micronutrients, can promote wound healing [15, 29, 33, 34]	1a	A
12	Supplement with hydrolysed collagen [33, 38]	1a	B
13	Obesity and overweight may be associated with delayed wound healing in patients with nutritional metabolic wounds (venous ulcers and diabetic foot ulcers) [16, 17]	5b	A
14	For diabetic foot ulcer patients with a body mass index of more than 24 kg/m [2], an appropriate weight loss of 3%–5% is recommended [17]	5b	A
15	All patients with diabetic foot ulcer should try to choose high‐quality carbohydrates, those with high nutritional density, high dietary fibre, vitamins and minerals, with less sugar, fat or salt [17]	1b	B
16	Improve food services to provide more dining options for patients [16]	1c	B
17	If adequate intake cannot be met even after active dietary intervention, utilise enteral or parenteral nutrition to meet the patient's needs for calories, protein, water and micronutrients [35, 36]	5b	A
*(iii) Nutrition education and counselling*			
18	All patients and their caregivers should receive nutrition care education	5b	A
19	Provide nutrition training for all health personnel [16]	5b	A
20	First nutrition education should involve nutritionists/dietitians providing patients and their caregivers with information on nutrition management through educational materials, allowing participants to ask questions [19, 39]	5b	A
21	Patients with chronic wounds should be divided into different groups or receive personalised nutrition counselling. The nutritionists/dietitians should explain different food types and give examples of the most common food choices for each food item [39]	1c	A
22	First nutritional education and counselling typically last 10–30 min	1c	A
23	Nutrition counselling should take place every 4 weeks for approximately 5 min. Healthcare workers address potential problems and encourage patients to continue to choose healthier foods [39]	5b	A
24	Patients with diabetic foot ulcers should choose appropriate diets, such as Mediterranean dietary pattern, low carbohydrate dietary pattern, low‐fat dietary pattern, high dietary fiber dietary pattern [17]	5b	A
25	Patients receiving nutritional therapy should continue to monitor their nutritional status and increase the frequency of nutritional follow‐up after discharge. Nutritional follow‐ups include daily nutrition diaries, weight changes and alterations in the body's metabolic composition [16, 17]	5b	A
*(iv) Multidisciplinary teamwork*			
26	Patients who are malnourished or at risk of malnutrition develop and implement a personalised nutrition treatment plan using a multidisciplinary approach. This multidisciplinary team should include doctors, nurses, nutritionists, pharmacists and administrators [16, 17]	5b	A

## 4 Discussion

It is crucial for the positive health outcomes of patients with chronic wounds to uphold proper nutrition [5, 7]. However, there is currently a lack of systematic and comprehensive clinical nutrition management practices for patients with chronic wounds in China. Therefore, based on the best evidence, this study developed a nutrition management guidance flowchart for patients with chronic wounds. It is clearly comprehensible, easily embraced by medical personnel and patients and promotes scientific, standardised and normalised nutrition management among patients with chronic wounds in clinical settings.

### 4.1 Nutrition Assessment and Diagnosis

In the process of nutrition management for patients with chronic wounds, first, nutrition screening is a simple test that provides a primary nutrition diagnosis. Given its widespread usage, clinical workload and patient cooperation, NRS‐2002 was recommended for nutrition screening [38, 40]. Individuals with chronic wounds who have an NRS score of 3 or higher may benefit from further nutrition education or a nutrition care plan, although this does not necessarily indicate a requirement for nutritional therapy. It is necessary for medical staff to perform nutritional screening a week after admission [41]. It is recommended that healthcare professionals conduct nutrition assessments and establish secondary nutrition diagnoses using PG‐SGA among chronic wound patients at a nutritional risk [17]. When compared to other nutrition assessment instruments, PG‐SGA, which consists of patient self‐assessment and assessment by healthcare professionals [41], could enhance patient involvement, fostering improved collaboration between doctors and patients in nutritional care. Importantly, PG‐SGA relies more on individual subjective judgements of users than specific measurements [41]. Therefore, users (patients and doctors) must receive appropriate training in the standardised use of PG‐SGA to ensure accurate implementation. Furthermore, evidence has shown that MNA is a reliable tool to assess the decrease in appetite and dietary intake in elderly patients [42], with a good predictive value for short‐term weight loss, making it the preferred tool for determining the nutritional condition of older patients with chronic wounds. Following a malnutrition diagnosis in a patient with chronic wounds, a comprehensive nutrition assessment is necessary to identify the tertiary nutritional diagnosis, encompassing the type and causes of malnutrition, and organ dysfunction. After reviewing the available data [16, 17, 38], we developed an assessment checklist focusing on the details of the comprehensive nutrition assessment (see Evidence Item 3), encompassing dietary habits, autonomic feeding skills, medical assessments, pathological factors, laboratory biochemical results and psychiatric conditions.

Healthcare workers should complete nutrition screening, primary assessments, and comprehensive nutrition assessments for patients with chronic wounds sequentially, identify different levels of nutrition diagnosis and adjust nutrition management strategies in a timely manner. The evidence level for nutritional assessment and diagnosis is 5b, the recommendation grade is A and there is insufficient evidence regarding the timing, frequency and effectiveness of nutrition assessments for patients with chronic wounds.

### 4.2 Nutrition Care Plans

Based on the evidence at hand, patients with chronic wounds who are at nutritional risk should consume a minimum of 30–35 kcal/kg of energy and 1.25–1.5 g/kg of protein per day to promote tissue regrowth and healing [17, 38]. Furthermore, such patients characterised by skin damage and ongoing wound exudation may experience a continuous depletion of nutrients. Studies have shown that patients with wounds producing excess exudate may experience nitrogen loss, equivalent to approximately 6 g of protein per day [10]. Thus, if the standard diet for patients with chronic wounds does not satisfy the nutritional requirements, nutritional treatments (e.g., oral nutritional supplements, enteral nutrition and parenteral nutrition) should be provided with at least 4 weeks between meals [29, 37]. Although some interventional studies have explored the importance of certain micronutrients (e.g., Vitamins A and D, Multivitamin B and zinc) in promoting wound healing among patients with chronic wounds [31, 32, 43], our research group provides the following considerations when offering guidance on specific micronutrient interventions. On the one hand, a related survey has reported deficiencies in micronutrients in this group of diseases [28]. Some positive effects of supplementing certain micronutrients in patients with chronic wounds may be attributed to the absence of these micronutrients in these populations. On the other hand, some reports have suggested that the supplementation of specific micronutrients led to various adverse effects in patients [44, 45]. Hence, it is recommended to closely monitor the levels of micronutrients and appropriately supplement them to populations with deficiencies under the supervision of an expert doctor and nutritionist. Furthermore, there is still a lack of evidence on nutrition care plans for patients with chronic wounds. First, data on consumption patterns, supplement amounts and the safety of various micronutrients are scarce, and more investigations are needed to assess their effectiveness in different subgroups with wounds. Second, chronic wounds caused by trauma and infection tend to be larger, take longer to heal and may require more nutrients compared to those with other aetiologies, the nutrient requirements of these populations are not well‐supported by evidence. We look forward to further research in this area.

### 4.3 Nutrition Education and Counselling

Caregivers play an increasingly important role in providing nutrition care for patients with chronic wounds. Previous research has demonstrated that caregivers shoulder 70% of the complex responsibility for managing chronic illnesses [46]. Nutrition education is advised for all patients with chronic wounds and their caregivers, thereby promoting their health literacy and the development of more effective nutrition management skills [47]. In addition, the lack of nutritional knowledge among specialist doctors and nurses also hinders the provision of higher‐quality nutrition care [48]. Thus, it is recommended that nutrition care training is provided for all healthcare workers. Some studies have indicated that patients' food preferences are changing, leading to a marked decrease in protein and fat intake [19, 49]. Accessible evidence has emphasised the importance of nutrition counselling by nutritionists to meet patients' nutritional needs based on dietary preferences [17]. Moreover, in clinical practice, personalised dietary counselling can provide guidance and help patients incorporate healthy foods into their daily lives [19], rather than offering generic advice. The timing, frequency, utilisation of relevant visual aids and patient interaction are crucial elements to guarantee the efficacy of personalised nutrition counselling education.

### 4.4 Multidisciplinary Teamwork

A multidisciplinary team of doctors, nurses, nutritionists, pharmacists and administrators can effectively deliver high‐quality nutrition care [50]. However, the responsibilities of members of multidisciplinary teams are not clearly defined. In clinical practice, it is recommended that physicians and nutritionists should complement each other to provide more personalised and continuous nutrition treatments [17]. Nurses should not only perform nutrition screening but also monitor patients' nutrition needs, food intake and tolerance and continuously interact with patients and their caregivers [51]. They also act as a bridge between doctors, nutritionists, patients and paramedics. Therefore, we should give full play to nurses' initiative and establish a multidisciplinary team model led by them. Comorbidities and multi‐medicine use are often associated with the development of malnutrition among patients with chronic wounds [22], while there is relatively little research on nutritional medication management. We recommend that pharmacists/clinicians use appropriate drug screening criteria to review drugs for those at nutritional risk or malnourished to reduce the incidence of multiple medication use.

## 5 Limitations

First, although this study provided a comprehensive overview of evidence‐based nutrition management programmes for patients with chronic wounds, differences in patients' geographical location, ethnicity and cultural background could have influenced the results. Secondly, the research was limited to English and Chinese databases, and publications in other languages were excluded. Additionally, the evidence should be updated in future studies, and appropriate evidence should be selected based on feasibility, suitability, efficacy and clinical practice to facilitate the implementation of such evidence. Finally, the applicability, benefit or efficacy of this nutrition management guidance flowchart for patients with chronic wounds based on evidence need to be verified [20].

## 6 Conclusion

In this work, the best evidence related to nutrition management in patients with chronic wounds was summarised, and a nutrition management guidance flowchart based on this best evidence was constructed to provide references for medical staff in guiding nutrition management for patients with chronic wounds. However, the associated literature was limited, and the quality was relatively low. Thus, more high‐quality research is needed in the future to focus on specific personalised nutrition management programmes for patients with chronic wounds.

## Conflicts of Interest

The authors declare no conflicts of interest.

## Data Availability

The data that support the findings of this study are available from the corresponding author upon reasonable request.
